# Editorial: Insights into the functional role of extracellular vesicles with specific cell types following infection and inflammation

**DOI:** 10.3389/fimmu.2023.1260310

**Published:** 2023-08-04

**Authors:** Bin Gong, Yang Jin, Tais Berelli Saito

**Affiliations:** ^1^Department of Pathology, University of Texas Medical Branch, Galveston, TX, United States; ^2^Pulmonary and Critical Care Medicine Division, Department of Medicine, Boston University, Boston, MA, United States; ^3^Laboratory of Bacteriology, Division of Intramural Research, NIAID-NIH, Hamilton, MT, United States

**Keywords:** extracellular vesicles, cell types, infection, inflammation, functional role

This Research Topic has provided valuable contributions to our understanding of the diverse roles and potential therapeutic applications of extracellular vesicles (EVs) in the context of infection and inflammation. The seven articles published in this Research Topic encompassed a range of original research and review articles, shedding light on various aspects of EV biology and their implications in disease pathogenesis and therapeutic strategies.

In the original research article “*Exosomally Targeting microRNA23a Alleviates Microvascular Endothelial Barrier Dysfunction Following Rickettsial Infection*,” Changcheng Zhou et al. focused on the impact of microRNA23a (miR23a)-enriched exosomes derived from rickettsial-infected endothelial cells (ECs) on microvascular endothelial barrier dysfunction. The findings demonstrated that miR23a delivered by exosomes impaired barrier function in recipient endothelial cells, highlighting the potential of exosome-based therapies for improving vascular barrier function during bacterial infection and inflammation.

In the original research article “*Extracellular vesicles from Listeria monocytogenes-infected dendritic cells Modulate the Innate Immune Response*,” Izquierdo-Serrano et al. demonstrated that EVs derived from Listeria-infected dendritic cells play a role in communicating with neighboring, uninfected dendritic cells and promoting antiviral responses. These findings highlight the importance of EVs in coordinating immune responses during infection.

In the original research article “*Circulating Brain-Derived Extracellular Vesicles Expressing Neuroinflammatory Markers are Associated with HIV-Related Neurocognitive Impairment*,” Marques de Menezes et al. investigated the role of EVs in HIV-related neurocognitive impairment. The study demonstrated that EVs expressing neuroinflammatory markers were associated with cognitive deficits in individuals with HIV, suggesting their potential as biomarkers of neuronal injury in HIV infection. Furthermore, the study revealed an interaction between circulating platelet EVs and monocyte activation, implicating their involvement in the pathogenesis of HIV-related cognitive impairment.

In the original research article “*A crucial exosome-related gene pair (AAMP and ABAT) is associated with inflammatory cells in intervertebral disc degeneration*,” Ren et al. identified a crucial exosome-related gene pair (AAMP and ABAT) associated with intervertebral disc degeneration (IDD), suggesting their potential as biomarkers for diagnosing IDD. This study emphasized the importance of investigating exosome-related genes and competing endogenous RNAs to enhance the understanding of IDD mechanisms.

In the original research article “*Circulating extracellular vesicles are associated with the clinical outcomes of sepsis*,” Li et al. revealed that EVs, specifically those carrying caspase-1 and miR-126, were linked to vascular injury and the development of acute respiratory distress syndrome (ARDS) and acute renal failure (ARF). These observations highlight the potential of EVs as prognostic biomarkers and therapeutic targets in sepsis.

In the review article “Immune Cell-Derived Extracellular Vesicles in Pathogenic Infections,” Keshtkar et al. explored recent studies on immune cell-derived EVs in viral, bacterial, fungal, and parasitic infections. The cargo carried by EVs, including miRNAs, mRNAs, and proteins, appears to mediate the antimicrobial process, suggesting the potential of immune cell-derived EVs as therapeutic agents.

In review article “*Translating extracellular vesicle packaging into therapeutic applications*”, Ozkocak et al. discussed the therapeutic potential of EVs as drug delivery platforms and their cargo loading mechanisms. Understanding how biomolecules are packaged into EVs is crucial for harnessing their therapeutic capabilities and expanding their clinical applications.

Overall, the articles published in this Research Topic have significantly contributed to advancing our knowledge of the functional roles of EVs in specific cell types following infection and inflammation. The findings have shed light on the complex interplay between EVs and recipient cells, highlighting their diagnostic, therapeutic, and biomarker potential in various diseases. Further exploration of EV biology and their specific cargo sorting mechanisms will pave the way for the development of novel therapeutic strategies and diagnostic tools in the field of immunology and infectious diseases.

## Author contributions

BG: Writing – original draft, Writing – review & editing. YJ: Writing – original draft, Writing – review & editing. TS: Writing – original draft, Writing – review & editing.

